# Optimal Arrangements and Local Anisotropy of {100} Guinier–Preston (GP) Zones by Parametric Dislocation Dynamics (PDD) Simulations

**DOI:** 10.3390/ma17205076

**Published:** 2024-10-18

**Authors:** Haiwei Zheng, Jianbin Liu, Shinji Muraishi

**Affiliations:** 1 Department of Materials Science and Engineering, Tokyo Institute of Technology, Tokyo 152-8550, Japan; 2 Midea Group Co., Ltd., Foshan 528311, China

**Keywords:** dislocations, dynamics, microstructures

## Abstract

Stress-oriented precipitation and the resulting mechanical anisotropy have been widely studied over the decades. However, the local anisotropy of precipitates with specific orientations has been less thoroughly investigated. This study models the interaction between an edge dislocation source and {100} variants of Guinier–Preston (GP) zones in Al-Cu alloys using the parametric dislocation dynamics (PDD) method. Concentric geometrically necessary dislocation (GND) loops were employed to construct a line integral model for thin platelets. The simulations, conducted with our self-developed code based on Green’s function method and Eshelby inclusion theory revealed distinct strengthening behavior along the strong and weak directions for 60° GP zones, demonstrating anisotropic strengthening from the perspective of elastic interactions. Furthermore, the optimal inclined arrangement of the GP zone array was determined through elastic energy calculations, and these results were corroborated by TEM observations.

## 1. Introduction

Precipitation hardening is a common strategy employed to enhance the yield strength and hardness of heat-treatable alloys. During the aging process, successive transformations from metastable phases to the stable phase lead to variations in the mechanical properties of alloys. In the early stages of precipitation, metastable phases maintain a coherent or semi-coherent relationship with the matrix lattice. Differences in lattice constants and elastic moduli between precipitates and the matrix induce coherency strain fields. According to the Mori–Tanaka homogenization theory [1], the actual stress is composed of the average stress plus the locally fluctuating stress, with the average stress vanishing in the matrix. Consequently, coherency stress around precipitates will interact locally with dislocations, affecting the flow stress level.

The generally accepted precipitation sequence at ambient temperature in the Al-Cu alloy follows this order: GP zones (GP1 phase), θ″ phase (GP2 phase), θ′ phase, and θ phase. This sequence plays a crucial role in determining mechanical properties and strengthening mechanisms of the material.

Guinier [2] and Preston [3] first identified these zones in 1938, where copper atoms segregate on ${\left\{100\right\}}_{Al}$ crystal planes to form Cu-rich monolayers, with ${\left\{100\right\}}_{Cu}$ parallel to ${\left\{100\right\}}_{Al}$ (i.e., ${\left\{100\right\}}_{Cu}//{\left\{100\right\}}_{Al}$). Kamijo [4] utilized the finite element method to evaluate the strain energy levels of solute copper atoms and GP zones. Their findings suggest that the strain energy of copper atoms is highest in a solid solution, while the overall strain energy of the system decreases as the radius of GP platelets increases. Consequently, the formation of GP zones in an Al-Cu alloy primarily occurs through the relaxation of strain energy surrounding solute copper atoms. Wolverton [5] conducted first-principles calculations to predict the equilibrium shape of GP zones in Al-Cu. Gerold [6] and Matsubara [7] performed structural analyses and determined a compressive strain of approximately 10% along the disk’s normal direction.

In 1978, Eto [8] verified the stress-oriented effect of platelet precipitates in an Al-Cu alloy. Muraishi [9] subsequently reported the strengthening anisotropy resulting from various orientations of platelets. This strengthening anisotropy, caused by the different orientations of precipitates, has gained significant attention among researchers. Singh [10] explored the hardening effects of a single GP zone, which exhibits either 0° or 60° between the dislocation Burgers vector and the disk/primary plane intersection line.

In 1999, Gladman [11] proposed equations delineating the strengthening effects of “soft” deformable particles and “hard” nondeformable particles, where the distinction between “soft” and “hard” is contingent upon the critical particle size. Microstructural observations have corroborated various interaction mechanisms, such as dislocation shearing a soft particle while bypassing a hard one through Orowan looping or cross-slip mechanisms. These mechanisms are supported by molecular dynamics (MD), a valuable tool at the microscale for simulating interactions between dislocations and precipitates, including soft GP zones. Esteban-Manzanares [12] conducted atomistic modeling, confirming that dislocations shear GP zones in Al-Cu. Krasnikov [13] investigated the effects of GP zone diameters and the number of layers in the θ″ phase (GP2 phase) on the flow stress level.

During the 1960s, Brown [14], Bacon [15], and Foreman [16] introduced the methodology of dislocation dynamics (DD). Santos-Güemes [17] utilized discrete dislocation dynamics (DDD) to study dislocation–θ′ interactions in an Al-Cu alloy. Ghoniem [18] introduced parametric dislocation dynamics (PDD), wherein dislocation segments are represented as parametric space curves. DD has since become a valuable tool for simulating interactions between dislocations and precipitates, as well as dislocation–dislocation interactions, at the mesoscale.

In our previous studies, we replicated the elastic fields of the plate-shaped Ω phase in the Al-Cu-Mg-Ag alloy [19] and the needle-shaped β″ phase in the Al-Mg-Si alloy [20] using surface integral models based on the Eshelby inclusion theory. PDD simulations were employed to investigate several factors influencing precipitation strengthening levels. In this study, we successfully constructed a line integral model of GP zones using concentric GND loops and obtained the elastic fields of GP zones. The elastic interaction energy between GP platelets was analyzed, facilitating the determination of optimal arrangements. We further investigated the strengthening effects of {100} GP zone/arrays by conducting GP–dislocation interactions using the PDD method. The primary objective is to explore the optimal arrangements of {100} GP zones and confirm the local anisotropy induced by various precipitate orientations. The significance of this study lies in deepening the understanding of GP zones from the perspective of elastic interactions, which contributes to the design of anisotropic aluminum alloys.

## 2. Methods and Theory

### 2.1. Simulation Model

According to the Eshelby inclusion theory [21,22], GP zones composed of copper atoms can be regarded as inhomogeneous inclusions. Previous experiments [6] reported a constraint strain around the GP platelets $\varepsilon_{ii}^{C}=\left(0,\;0,-0.1\right)$. The equivalent eigenstrain $\varepsilon_{ii}^{*}$ can be calculated using the following equation [23]:

(1) $$\varepsilon_{ij}^{C}=S_{ijkl}\varepsilon_{kl}^{*},$$

where S is the Eshelby tensor of an ellipsoid with an aspect ratio of 0.015. The reason for selecting this aspect ratio value is elucidated in the Results Section. The resulting equivalent eigenstrain $\varepsilon_{ii}^{*}=\left(-0.01,-0.01,-0.09\right)$ closely matches the constraint strain. Therefore, we adopt the eigenstrain of GP zones to be equivalent to the constraint strain ($\varepsilon_{ii}^{*}=\varepsilon_{ii}^{C}$).

The uniaxial eigenstrain $\varepsilon_{33}^{*}$ is expressed by displacement normal to the platelet $u_{3}^{*}$ as follows:

(2) $$\varepsilon_{33}^{*}=\frac{a_{GP}-a_{Al}}{a_{Al}}=\frac{u_{3}^{*}}{a_{Al}}=-0.1,$$

**Figure 1:**
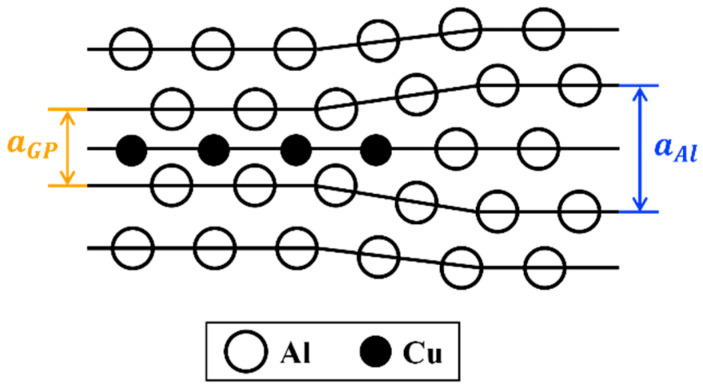
Schematic of a GP zone embedded in the Al matrix phase.

Figure 1 plots the schematic of the GP zone. The $a_{Al},\;a_{GP}$ are lattice constants of the aluminum matrix and GP zone along the [100] direction, respectively. With $a_{Al}=0.404\;\mathrm{nm}$, the lattice constant of the GP zone is calculated to be $a_{GP}=0.363\;\mathrm{nm}$ and displacement $u_{3}^{*}=-0.040\;\mathrm{nm}$.

For comparison, the mean value of the lattice constant $\bar{a}$ for one layer of aluminum atoms and one layer of copper atoms is calculated to be

(3) $$\bar{a}=\frac{a_{Al}+a_{Cu}}{2}=0.382\;\mathrm{nm},$$

The experimentally obtained $a_{GP}$ is a little bit smaller than the mean value $\bar{a}$, suggesting that vacancies exist in the copper atom layer.

In this study, each GP zone comprises 10 concentric GND loops, utilized solely for constructing the numerical model and lacking physical significance. Total displacement on the disk plane is expressed as the sum of loop Burgers vectors [24], as given by the following equation:

(4) $$u_{3}^{*}=b_{3}^{*}=\overset{N=10}{\underset{n=1}{\sum }}b_{3}^{n}=-0.040\;\mathrm{nm},$$

Given that all GND loops are placed on the same plane, the Burgers vector of the n-th loop, $b^{n}$, is assigned based on the loop area.

**Figure 2:**
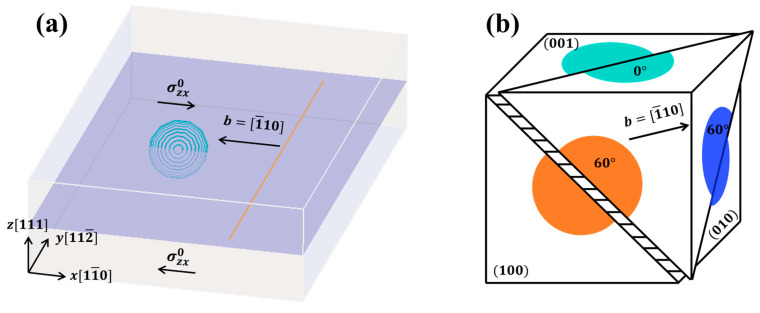
(**a**) Schematic of the edge dislocation (orange line) interacting with single (001) GP zone (turquoise concentric loops). (**b**) Geometric relationships between the $\left(111\right)\left[\bar{1}10\right]$ slip system and {100} GP platelets.

Figure 2a illustrates the primary slip system as $\left(111\right)\left[\bar{1}10\right]$, with the x, y, and z axes aligned with the crystal orientations of $\left[1\bar{1}0\right],\;\left[11\bar{2}\right],\;\left[111\right]$, respectively. The matrix phase is defined as face-centered cubic (fcc) Al, with Young’s modulus E=70 GPa, shear modulus μ=27 GPa, Poisson’s ratio ν=0.3, and Burgers vector $b=\left[\bar{1}10\right]$ and $\left|b\right|=0.286\;\mathrm{nm}$. We use Green’s function method for calculations and there is no boundary condition applied in the current simulation model. The characteristic volume is set to calculate the strain rate, with a value of $1\times 10^{6}\;{\left|b\right|}^{3}\;\left(x=40\left|b\right|,\;y=500\left|b\right|,\;z=50\left|b\right|\right)$ [25]. The radius and thickness of GP zones are $15\left|b\right|\;$(4.29 nm) [26] and $1.27\left|b\right|\;$(0.36 nm), respectively. The volume fraction of the GP zone was fixed at 0.09%. The length of edge dislocation (orange line) and the cut-off length (core radius) are $494\left|b\right|$ and $0.5\left|b\right|$, respectively. The dislocation source with fixed ends will expand under the applied shear stress $\sigma_{zx}^{0}$.

Previous studies commonly categorized {100} GP zones into two types based on the angle between the dislocation Burgers vector and disk/primary plane intersection line. As depicted in Figure 2b, the Burgers vector $b=\left[\bar{1}10\right]$ aligns at 0 degrees with the intersection line of the (001) disk, whereas for the (010) and (100) disks, it aligns at 60 degrees. This implies that the (010) and (100) orientations are crystallographically equivalent in the $\left(111\right)\left[\bar{1}10\right]$ slip system. However, in this simulation, the dislocation only approaches from one side of the precipitates. Hence, it is necessary to consider three variants of the {100} disks.

### 2.2. Stress Components

The simulations were conducted using our self-developed code, written in Fortran and C, and the results were visualized with GiD v13.0 The algorithm implemented in the code is described as follows: According to the PDD method, dislocation lines are discretized into a series of curve segments connected by nodes. The total stress $\sigma_{ij}^{t}$ acting on each node is expressed as follows:

(5) $$\sigma_{ij}^{t}=\sigma_{ij}^{0}+\sigma_{ij}^{in}=\sigma_{ij}^{0}+\sigma_{ij}^{\Omega }+\sigma_{ij}^{D},$$

where $\sigma_{ij}^{0}$ is the applied stress, the internal stress $\sigma_{ij}^{in}$ comprises stress from both precipitates $\sigma_{ij}^{\Omega }$ and dislocations $\sigma_{ij}^{D}$.

A constant total strain rate ${\dot{\varepsilon }}_{ij}^{t}=10^{3}\;s^{-1}$ was specified. The applied stress at a certain time step is expressed as follows:

(6) $$\sigma_{ij}^{0}=C_{ijkl}\left(\varepsilon_{ij}^{t}-\varepsilon_{ij}^{p}\right),$$

where $C_{ijkl}$ is the stiffness of the matrix phase. The plastic strain $\varepsilon_{13}^{p}$ is acquired from the glide motion of dislocations as follows:

(7) $$\varepsilon_{13}^{p}={\dot{\varepsilon }}_{13}^{p}\cdot \Delta t=\frac{b\Delta A}{V},$$

where ΔA is the swept area of dislocations on the primary plane, Δt is the motion time, and V is the characteristic volume.

In this study, both precipitate stress $\sigma_{ij}^{\Omega }$ and dislocation stress $\sigma_{ij}^{D}$ are calculated using the line integral from of Green’s function:

(8) $$\sigma_{ij}^{in}\left(x\right)=C_{ijkl}\int \in_{lnh}C_{pqmn}G_{kp,q}\left(x,x^{\prime }\right)b_{m}\xi_{h}dL\left(x^{\prime }\right),$$

where x, x' denote the field point and source point, respectively. $\in_{lnh}$ is the permutation tensor, $G_{ij,k}$ is the first derivative of Green’s function, $b_{i}$ is the Burgers vector, and $\xi_{i}$ is the tangent vector of dislocation nodes.

A rotation matrix $A^{R}$ was employed to transform the elastic fields of {100} disks from local coordinates to global coordinates:

(9) $$\sigma_{ij}=a_{ik}a_{jl}\sigma_{kl}^{'},$$

where $a_{ik}$ is the component of the rotation matrix, which varies with the orientations of precipitates. Details of the transformation matrix can be found in our previous work [20].

The Peach–Koehler (PK) force $f_{m}$ acting on nodes can be calculated as follows:

(10) $$f_{m}=\in_{jmn}\sigma_{ij}^{t}b_{i}\xi_{n},$$

Both cross-slip and annihilation mechanisms were considered, as they significantly influence dislocation bypassing precipitates. The secondary slip plane was defined as the $\left(11\bar{1}\right)$ plane. At each time step, the Peach–Koehler force components $f_{g}$ on the primary slip plane and $f_{cs}$ on the secondary slip plane are compared and cross-slip occurs when $f_{cs}>f_{g}$. Regarding the annihilation reaction, it occurs when two nodes possess the same Burgers vector and opposite tangent vector, and when the distance between them is less than $20\left|b\right|$.

### 2.3. Energy Analysis

The mutual interaction between two dislocation loops can be determined by performing a volume integral of the energy density arising from the stress field of one loop, acting on the strain field of the other loop, as described below:

(11) $$E_{I}=\int \sigma_{ij}^{\left(1\right)}e_{ij}^{\left(2\right)}dV,$$

where $\sigma_{ij}^{\left(1\right)}$ is the elastic stress field from the first dislocation loop and $e_{ij}^{\left(2\right)}$ is the elastic strain tensor originating from the second one. After derivation, deWit [27] provided a simple double line integral formulation for the interaction energy:

(12) $$E_{I}=-\frac{\mu b_{i}^{\left(1\right)}b_{j}^{\left(2\right)}}{8\pi }\oint \oint \left[R_{,kk}\left(dl_{j}^{\left(2\right)}dl_{i}^{\left(1\right)}+\frac{2\nu }{1-\nu }dl_{i}^{\left(2\right)}dl_{j}^{\left(1\right)}\right)+\frac{2}{1-\nu }\left(R_{,ij}-\delta_{ij}R_{,ll}\right)dl_{k}^{\left(2\right)}dl_{k}^{\left(1\right)}\right],$$

where R is the modulus of vector connecting the source point to the field point. As we mentioned before, the GP zone is composed of ten concentric dislocation loops, and the double line integral is applied over pairs of these dislocation loops.

The change in Gibbs free energy δG was assumed to be related to the works conducted by internal stress and applied stress as follows:

(13) $$\delta G=\delta E^{D}+\delta E^{P}+\delta P,$$

with

(14) $$\delta E^{D}=-\int \in_{jmn}\left(\underset{s\left(s\neq t\right)}{\sum }\sigma_{ij}^{D\left(s,t\right)}+\frac{1}{2}\sigma_{ij}^{D\left(t,t\right)}\right)b_{i}^{\left(t\right)}\xi_{n}\delta u_{m}^{\left(t\right)}dl,$$

(15) $$\delta E^{P}=-\int \in_{jmn}\left(\underset{p}{\sum }\sigma_{ij}^{\Omega \left(p,t\right)}\right)b_{i}^{\left(t\right)}\xi_{n}\delta u_{m}^{\left(t\right)}dl,$$

(16) $$\delta P=-\delta W=-\int \in_{jmn}\sigma_{ij}^{0}b_{i}^{\left(t\right)}\xi_{n}\delta u_{m}^{\left(t\right)}dl,$$

where $\delta E^{D},\;\delta E^{P},\;\delta P$ represent the variations in the dislocation interaction energy (including self-energy), the precipitate–dislocation interaction energy, and the potential energy, respectively. $\sigma_{ij}^{D\left(s,t\right)}$ and $\sigma_{ij}^{\Omega \left(p,t\right)}$ indicate the stress caused by the s-th dislocation segment and the p-th precipitate, acting on the t-th dislocation node.

### 2.4. Sample Preparation and Characterization

The copper content in heat-treatable Al-Cu alloys typically ranges from 2% to 6% by weight. This copper concentration significantly enhances the alloy’s strength and hardness, particularly after heat treatment processes, where the formation of precipitates further improves its mechanical properties. The as-cast aluminum ingot used in this study was supplied by Nikkei MC Aluminium Co., Ltd., and its chemical composition is listed in Table 1.

The ingot was homogenized at 525 °C for 48 h and subsequently hot-rolled into plates with a reduction rate of 50%. The sample was solution-treated at 525 °C for 4 h and quenched to room temperature using water. Subsequently, stress aging was carried out at 130 °C for 1 h using an oil bath (Yamato BOA201, Tokyo, Japan), while applying a compressive stress of 80 MPa with a constant load machine (Yonekura MFG, Osaka, Japan). This was followed by the second step of stress-free aging at 150 °C for 20 h. Thin foils for TEM observations were prepared by using twin-jet electropolishing (Struers TenuPlo-5, Ballerup, Denmark). The microstructure of the stress-aged specimen was characterized using a transmission electron microscope (TEM, JEOL JEM-3010 microscope, Tokyo, Japan) operating at an accelerating voltage of 200 kV.

## 3. Results

### 3.1. Single GP Zone

**Figure 3:**
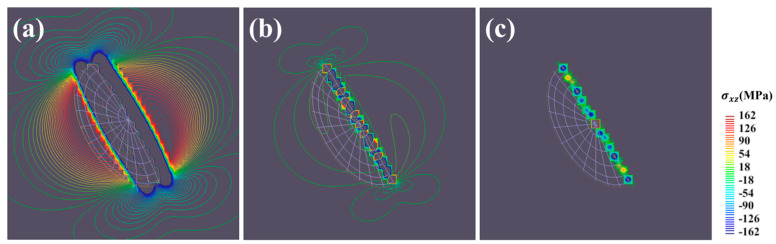
Contour lines of shear stress $\sigma_{xz}$ around (100) oblates on the (111) plane. The aspect ratio of the oblate equals (**a**) 0.1, (**b**) 0.01, and (**c**) 0.001.

The elastic fields of the oblate precipitate obtained through the surface integral model have been previously validated in our prior study [28] by comparing them with the analytical solutions provided by Eshelby. Figure 3 displays the contour lines of shear stress $\sigma_{xz}$ around (100) oblates with varying aspect ratios. A uniaxial eigenstrain of $\varepsilon_{33}^{*}=-0.1$ was applied to the oblates; misfit stress levels outside the oblates decrease as the thickness diminishes.

**Figure 4:**
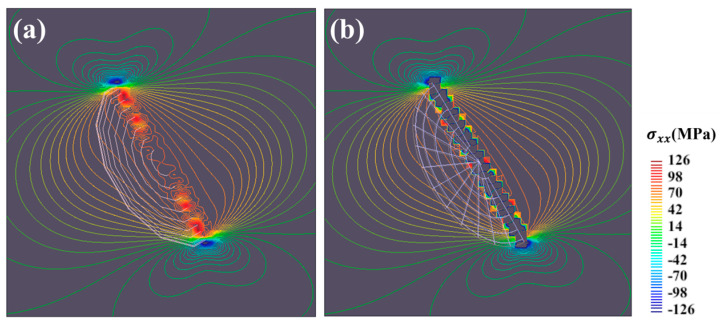
Contour lines of the normal stress component $\sigma_{xx}$ around the (**a**) (100) disk (line integral model) and (**b**) (100) thin oblate (surface integral model) with an aspect ratio of 0.015 on the (111) plane.

We built a line integral model with concentric GND loops tailored to replicate the elastic fields of the very thin GP zones. The results of elastic fields for a single loop [29] and interactions between the loop and a straight dislocation line [30] exhibit good consistency with the analytical solution provided by Kroupa. Misfit stresses outside the disk were validated by comparing them to those of thin oblates. Simulations suggested that the stress distributions outside the GP disk are similar to that of an oblate with an aspect ratio of 0.015. Figure 4 depicts the contour lines of normal stress $\sigma_{xx}$ around the (a) (100) disk (obtained using a line integral) and (b) (100) thin oblate (obtained using a surface integral) with an aspect ratio of 0.015. A positive stress distribution is obtained near the disk surface, which decreases with increasing distance from the center point and transforms to negative in the far field. Negative stress concentrations also appear at the edges of the precipitates. Only minor singularities (less than 151 MPa) are present around the loop lines, while serious singularities (up to 6067 MPa) are observed near the oblate surface. The boundary stresses cannot be determined by numerical integral because the second derivative of Green’s function causes $O\left(r^{-3}\right)$ singularity. However, stress at the boundary can be obtained through boundary traction and displacement calculations [24].

**Figure 5:**
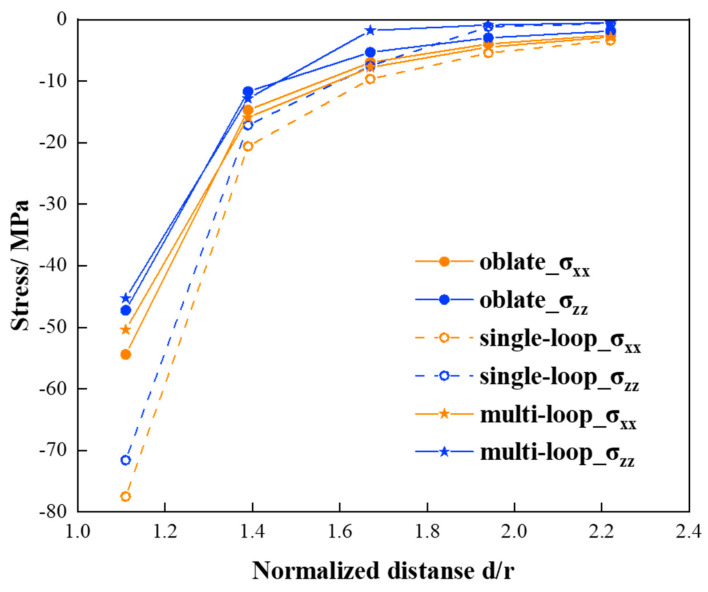
Normal stresses outside the (111) single/multi-loop disks and the (111) thin oblate on the (111) plane.

Figure 5 illustrates the normal stresses outside the (111) single/multi-loop disk and the (111) thin oblate. In the case of a multi-loop disk, distinct Burgers vectors were assigned to each loop based on their respective areas. The vertical axis in Figure 5 represents the normal stress level, while the horizontal axis indicates the normalized distance from the disk center, where $r=15\left|b\right|$. Negative normal stresses are observed outside the (111) precipitates, tapering off to zero as the normalized distance increases. The multi-loop disk (marked with a star) exhibits a similar stress level to the thin oblate (marked with a filled circle), whereas the single-loop disk (marked with a hollow circle) yields a higher stress level than the other two cases, especially in the area close to the disk. To conclude, the multi-loop model offers advantages in replicating the elastic fields of thin precipitates, as it entails fewer singularities and provides more accurate stress levels.

**Figure 6:**
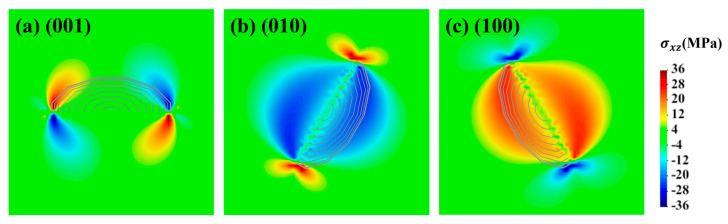
Contours of the shear stress component $\sigma_{xz}$ around the (**a**) (001), (**b**) (010), and (**c**) (100) GP zones on the (111) plane.

Figure 6 shows the contours of shear stress component $\sigma_{xz}$ around the (a) (001), (b) (010), and (c) (100) GP zones, which has an impact on the glide motion of edge dislocation. Stress distributions for the three orientations vary on the (111) plane. Regarding the (001) disk, positive and negative shear stress symmetrically distributes at the edge of the disk. On the other hand, the (010) disk generates negative stress fields around it, while positive stress concentrations appear at the edge of the disk. The scenario for the (001) disk is opposite to that of the (010) disk. From the perspective of average stress, the sum of the internal stresses is zero on a large enough scale; therefore, this study only discusses the local anisotropy.

**Figure 7:**
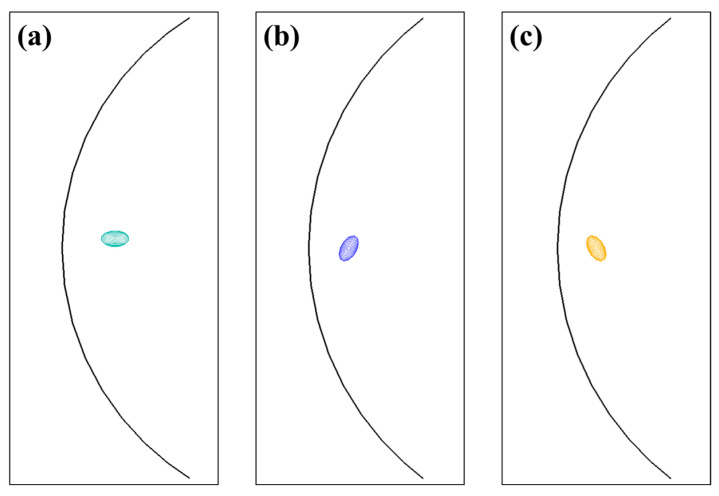
Snapshots after dislocation line shearing through the single (**a**) (001), (**b**) (010), and (**c**) (100) GP zones.

Snapshots in Figure 7 depict the dislocation line shearing through the single (001), (010), and (100) GP zones, respectively. The edge dislocation source was driven and bowed under the applied stress and subsequently sheared the disks without resorting to cross-slip or Orowan looping.

**Figure 8:**
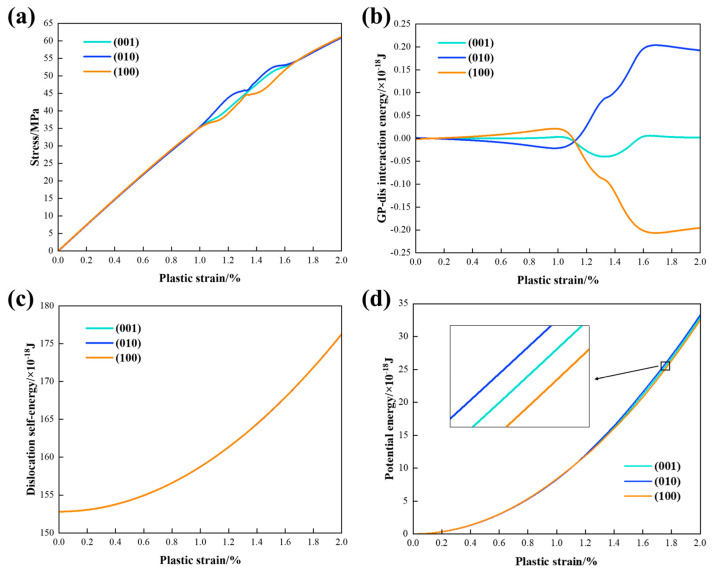
(**a**) Stress–plastic strain curves; (**b**) precipitate–dislocation interaction energies; (**c**) dislocation self-energies; (**d**) absolute potential energies of the edge dislocation interacting with single {100} GP zones.

Figure 8a presents the stress–plastic strain curves depicting the interaction of the edge dislocation with a single {100} GP zone. Expansion of the dislocation source is driven by the applied stress, which continues to increase until the dislocation source achieves the maximum curvature. A constant strain rate is maintained by adjusting the applied stress level as described in Equation (6) and precipitate shear stress $\sigma_{xz}$ acting on the dislocation nodes leads to variations in hardening rates. When dislocation nodes are far from the GP zone, far-field shear stress plays a significant role. The negative $\sigma_{xz}$ from the (100) disk (orange) retards dislocation glide motion and slightly elevates the flow stress level, whereas the positive $\sigma_{xz}$ from (010) disk (blue) facilitates dislocation glide, leading to a decrease in the flow stress. As some dislocation nodes approach the disk, the near-field shear stress becomes dominant. In this scenario, the negative $\sigma_{xz}$ generated by the (010) disk results in a higher flow stress, while the positive $\sigma_{xz}$ generated by the (100) disk leads to a lower stress level. When the plastic strain reaches 1.33%, dislocation sweeps across the opposite stress concentration at the edges of the (010) and (100) disks, resulting in opposing fluctuations in the curves. After shearing and leaving the GP zone, all the dislocation nodes re-enter the far-field stress region, where the hardening or softening effect is similar to the first stage. The flow stress level for the (001) case (turquoise) remains between the other two cases due to the symmetrically distributed stress field.

Figure 8b illustrates the corresponding curves of GP–dislocation interaction energy. The interaction energy of the (001) disk vanished after dislocation uniformly swept the symmetrically distributed elastic field, indicating a zero average shear stress near the (001) disk. For the (010) disk, the positive far-field shear stress assists the motion of dislocation glide, resulting in a slight decrease in the interaction energy. When the plastic strain reaches 1.00%, dislocation nodes enter the negative stress field around the (010) disk, leading to an increase in the interaction energy. The small plateau observed at approximately 1.33% of plastic strain is associated with the positive stress concentration at the edge of the (010) disk. Dislocation nodes exit the region of near-field stress at 1.67% of plastic strain, causing the interaction energy to decrease again due to the influence of far-field positive shear stress. The scenario for the (100) curve is the opposite of that observed for the (010) case.

The dislocation self-energy presented in Figure 8c was calculated based on the total length of the dislocation line. The dislocation line directly sheared the {100} disks without resorting to Orowan looping or cross slip. As a result, the dislocation self-energies remain consistent across all three cases and are three orders of magnitude higher than the interaction energies.

Figure 8d illustrates the absolute potential energy curves, representing the work done by applied stress. An intersection point is visible for the three curves when the plastic strain reaches 1.12%, corresponding to the intersection point of interaction energies in picture (b). Subsequently, the near-field shear stress creates gaps between different orientations, as depicted in the enlarged image. Results indicate that dislocation requires the highest energy to overcome retardation from the (010) disk, while it needs the lowest energy to shear the (100) disk, which assists dislocation glide through the coherency strain field.

### 3.2. Multiple GP Zones

The self-energy of a single GP zone $E_{GP}$ was estimated to be 1.51×10^−16^ J, which comprises interaction energies and self-energies of GND loops. If there are n GP zones, total interaction energy $E_{T}$ of loops calculated by Equation (12) comprises interaction energies $E_{Int}$ between GP zones and their self-energies as follows:

(17) $$E_{T}=E_{Int}+nE_{GP},$$

**Figure 9:**
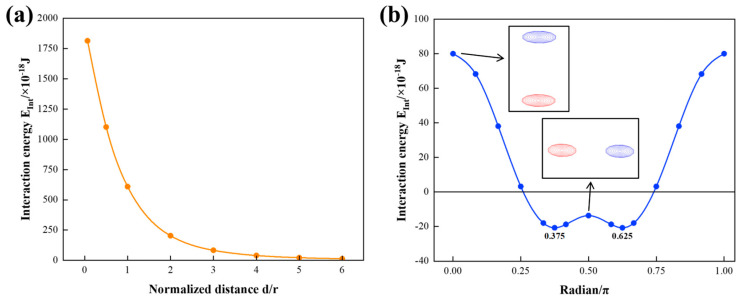
Elastic interaction energies of two (111) GP disks as a function of (**a**) normalized distance between the disk centers and (**b**) radian between the disk normal and the line connecting two disk centers.

Figure 9a illustrates the interaction energy $E_{Int}$ of two (111) GP disks as a function of the normalized distance between the disk centers. The interaction energy exhibits an inverse proportionality to the normalized distance and vanishes as the distance tends to infinity. In Figure 9b, the interaction energy is depicted as a function of the angle between the disk normal and the line connecting two disk centers, with the distance between the center points fixed at $60\left|b\right|$ (normalized distance d/r = 4). The energy variation reflects how the spatial distribution between GP zones affects their elastic interactions, without altering their crystallographic orientation or the distance between their centers. The configurations of two GP zones aligning at 0 and 0.5 π can be seen in the enlarged pictures. The interaction energy reaches its maximum when the two disks align with each other at 0 and π radians. Conversely, the minimum value of interaction energy is attained at 0.375 π and 0.625 π radians. When the radian is greater than about 0.25 π, the interaction energy is negative, indicating that the system status is stable. The radians corresponding to the lowest energy states might deviate slightly due to the non-perfect circular shape of the disk. This result implies that the elastic interaction energy between GP zones can be minimized by arranging them at specific angles.

**Figure 10:**
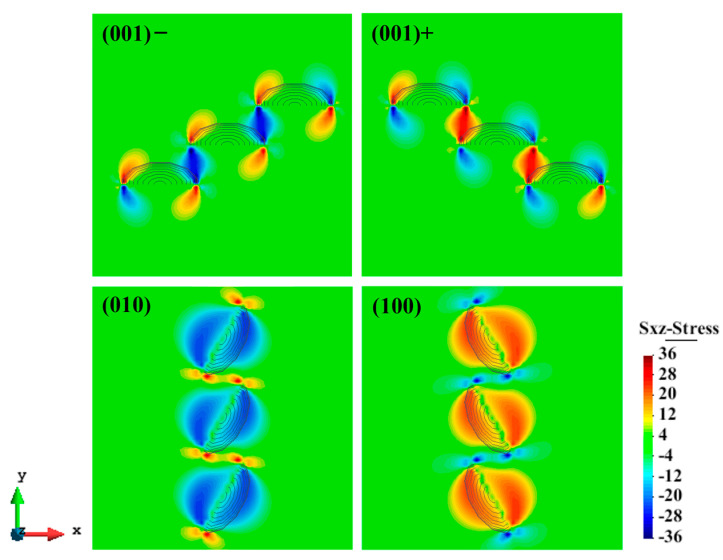
Optimal arrangements and contours of shear stress $\sigma_{xz}$ of (a) (001)−, (b) (001)+, (c) (010), and (d) (100) GP arrays on the (111) primary plane.

Based on the above interaction energy analysis, Figure 10 shows the optimal arrangements and the shear stress contours for the (a) (001)− (b) (001)+, (c) (010), and (d) (100) GP arrays. Regarding the (001) platelets, there are two types of optimal arrangements (001)− and (001)+, distinguished by the sign of superposed shear stress field. Similarly, the (010) and (100) platelets also arrange to achieve partial overlap of stress fields at the edge of disks. The configurations shown in Figure 10 suggest that GP platelets with the same orientation tend to overlap the elastic fields at the edge to minimize elastic interaction energy.

**Figure 11:**
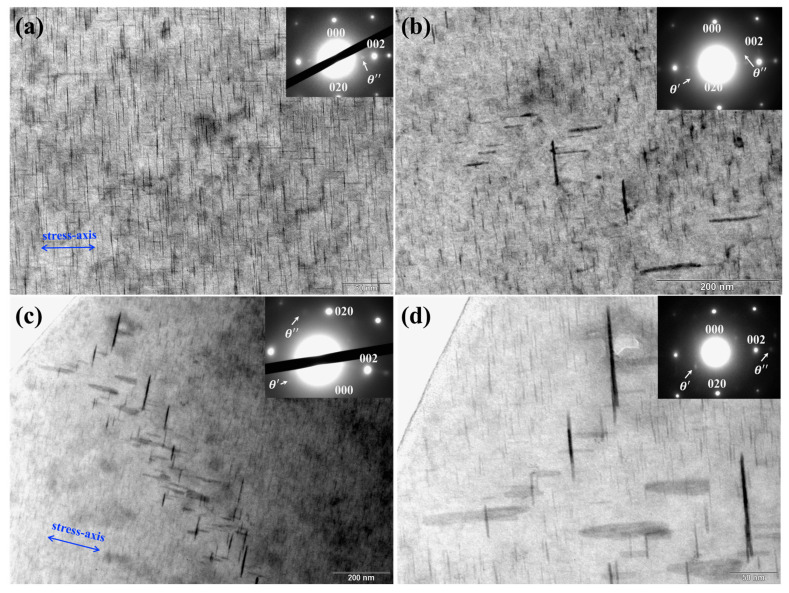
Diffraction patterns and bright-field TEM images taken along the ${\left[100\right]}_{Al}$ direction. (**a**) Homogenously distributed $\theta^{\prime \prime }$ phase. (**b**) Small-scale $\theta^{\prime }$ arrays. (**c**) Large-scale $\theta^{\prime }$ arrays. (**d**) $\theta^{\prime }$ arrays near the etch hole.

Figure 11 depicts TEM images and the corresponding electron diffraction patterns of the stress-aged specimens. Precipitates in Figure 11 were characterized to be a mixture of $\theta^{\prime \prime }$ phase and its subsequent $\theta^{\prime }$ phase. As the stress axis is close to ${\left[001\right]}_{Al}$, there is a higher number density of (001) platelets than that of the (010) platelets, suggesting that stress aging leads to preferential segregation of copper atoms on the crystal plane normal to the stress-axis, i.e., the stress-oriented effect. Picture (a) presents homogenously distributed $\theta^{\prime \prime }$ phase. Picture (b) shows small-scale $\theta^{\prime }$ arrays. Pictures (c) and (d) present large-scale arrays of $\theta^{\prime }$ platelets. The edge-up $\theta^{\prime \prime }$ phase and the $\theta^{\prime }$ phase appear as sticks and flat ovals, respectively. We can easily distinguish arrays of flat ovals arranged at the same angle. The inclined arrangements were consistent with the simulated configurations.

**Figure 12:**
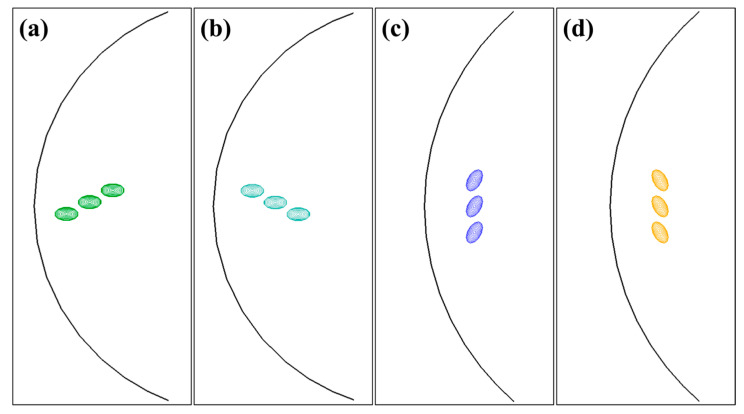
Snapshots after dislocation line shearing through the (**a**) (001)−, (**b**) (001)+, (**c**) (010), and (**d**) (100) GP zone arrays.

Snapshots in Figure 12 depict the dislocation line shearing through the (001)−, (001)+, (010), and (100) GP zone arrays. The edge dislocation source was driven and bowed under applied stress and subsequently sheared the disks without resorting to cross slip or Orowan looping.

**Figure 13:**
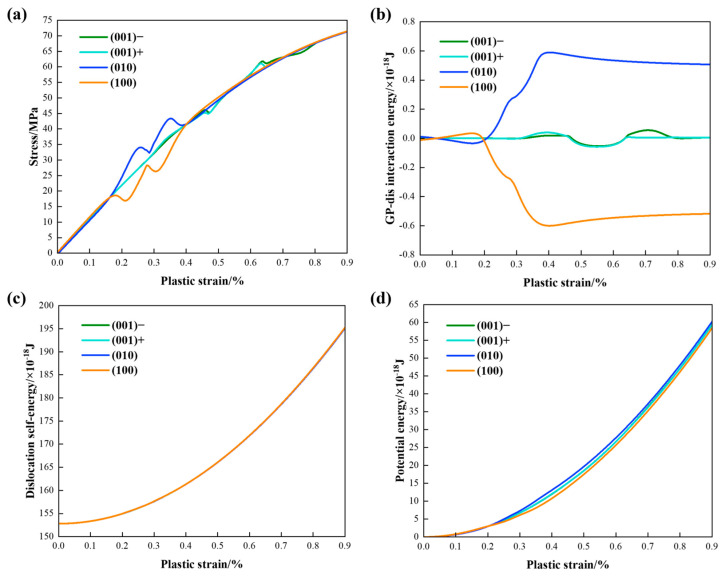
(**a**) Stress–plastic strain curves; (**b**) precipitate–dislocation interaction energies; (**c**) dislocation self-energies; (**d**) absolute potential energies of the edge dislocation interacting with {100} GP ar-rays.

In Figure 13a, the stress–plastic strain curves of the edge dislocation interacting with {100} GP zone arrays are presented. Since the volume fraction of the GP zone is fixed, the characteristic volume becomes three times that of the single GP zone case, resulting in a decrease in the hardening rate. When dislocation nodes are far from the GP zones, the far-field shear stress plays a crucial role. The negative $\sigma_{xz}$ from the (100) array retards dislocation glide motion and slightly elevates the flow stress level, whereas the positive $\sigma_{xz}$ from the (010) array assists dislocation glide, thereby reducing the flow stress. As some dislocation nodes approach the disks, the near-field shear stress becomes the dominant factor. In this scenario, the negative $\sigma_{xz}$ generated by the (010) array results in a higher flow stress, while the positive $\sigma_{xz}$ near the (100) array leads to a lower stress level. When the plastic strain reaches 0.29%, dislocation encounters enhanced opposite stress at the edges of platelets, resulting in opposing fluctuations on the curves. After shearing the GP zone arrays, all dislocation nodes re-enter the region of far-field stress, exhibiting a hardening or softening effect similar to the initial stage. Throughout most of the interaction process, the flow stress levels for the (001)− and (001)+ arrays remain between the (010) and (100) arrays, with fluctuations occurring when dislocation nodes pass through singularity regions.

In Figure 13b, the corresponding interaction energy curves of dislocation and GP zone arrays are presented. The interaction energies of the (001)− and (001)+ cases vanished after the dislocation evenly swept the symmetrically distributed elastic fields. Regarding the (010) array, the positive far-field shear stress assists dislocation glide motion and slightly decreases the interaction energy. Then, as dislocation nodes enter the negative stress field around the (010) array at approximately 0.17% plastic strain, the interaction energy increases. A small plateau emerges around 0.29% plastic strain, attributed to the positive stress concentration at the edge of (010) platelets. As dislocation nodes exit the region of near-field stress at 0.40% plastic strain, the interaction energy decreases again under the influence of far-field positive shear stress. Conversely, the situation for the (100) curve is exactly opposite that of the (010) case.

The dislocation self-energy presented in Figure 13c was calculated based on the total length of the dislocation line. The dislocation line directly sheared the {100} disk arrays without resorting to Orowan looping or cross slip. As a result, the dislocation self-energies remain consistent across all three cases and are three orders of magnitude higher than the interaction energies.

Figure 13d displays the absolute potential energy curves, representing the work done by applied stress. An intersection point is visible for the four curves when the plastic strain reaches 0.20%, corresponding to the intersection point of interaction energies in picture (b). Subsequently, the near-field shear stress creates larger gaps between different configurations than that of the single GP zone cases. Results indicate that the specific orientation of the GP zone array shows a similar trend as the single GP zone but with a more pronounced local hardening or softening effect.

Small oscillations can be seen in the curves in pictures (a) and (b), which are caused by the entry of dislocation nodes into the stress concentration regions near the edges of the (001) platelets. Specifically, the glide motion of a dislocation node is first promoted by the positive shear stress on the right side of the platelet, resulting in a slight decrease in applied stress to maintain a constant strain rate, as well as positive work done by the precipitates on the dislocation. As the dislocation node passes through the region of negative stress concentration, an inverse variation occurs. Once the dislocation uniformly sweeps through the region where the average shear stress is zero, the influence of the precipitate eventually vanishes from the perspective of elastic interactions. Additionally, the dislocation energy, calculated based on the total dislocation length, and the potential energy, derived from the total plastic strain, are not sensitive to the small stress oscillations mentioned above. As a result, no significant variations are observed in Figure 13c,d.

## 4. Discussion

Regarding the structure of GP platelets, Rioja [31] and Kashyap [32] observed evidence of spinodal decomposition in the early stages of GP zones, which are expected to form through concentration fluctuation. Hono [33] reported the existence of multi-layer GP zones with no spacing among the Cu-rich layers. Li [34] even observed an unreported phase where two Cu layers are separated by six Al layers. However, Wolverton [5] suggests that Cu bilayers and thicker {100} layers are energetically unfavorable and are not thermodynamically stable phases.

For a soft particle sheared by the dislocation, several effects may raise the stress level required for plastic yielding [11]. For example, the passage of a dislocation through a particle may produce an antiphase boundary or a stacking fault with associated energy. Lochte [35] and Hornbogen [36] proposed the interfacial energies of GP zone and θ″ phase to be 3 mJ/m^2^ and 50 mJ/m^2^, respectively. The interfacial energy of a GP zone in this study is 3.76×10^−19^ J, and the increase in interfacial energy for the 60° variants due to a single shear of dislocation is about 1.47×10^−20^ J, which is lower than the GP–dislocation interaction energy. Therefore, we took the elastic interactions as the only source of precipitation strengthening. However, chemical hardening mechanisms and complex short-range interactions should also be taken into account, which can be explored by MD simulations and will be the focus of our future work. Additionally, the obtained parameters are helpful in multi-scale modeling, including density functional theory (DFT), MD, DD, and finite element (FE). Singh et al. [37] performed atomistic-based hierarchical multiscale modeling to examine precipitation strengthening in an Al-Cu alloy. Bertin et al. [38] introduced a data-driven approach in which data obtained from large-scale MD simulations of crystal plasticity were employed to train the graph neural network (GNN) and model the dislocation mobility function. Gutiérrez-Menchaca et al. [39] have reported enhanced mechanical properties of silica glass and utilized the FE method to simulate large-scale elastic interactions involving nanoparticles.

Some research works have reported dissolution in Al-Cu [40] and Al-Mg-Si [41] alloys and phase transformations [42] of precipitates caused by multiple dislocation cuts, which is not applicable to this simulation. A previous experiment [9] indicated that only a very small number of dislocations cut through a single GP zone on one slip plane, judging from the extremely small scale of GP zones and the spacing between activated slip planes. In that case, we kept the elastic field of GP zones unchanged during and after the single dislocation shearing.

With a given uniaxial eigenstrain, the misfit stress level of the precipitate decreases with decreasing thickness. Despite assigning a mismatch strain of 10% normal to the disk plane, the stress level is lower than expected due to the extremely thin thickness of the GP zone. Numerical results of the coherency strain field for thick oblates with a uniaxial eigenstrain can be found in our previous article [19]. According to calculations by Lee [43], the strain energy in an Al-4wt.% Cu alloy is minimized when the ellipsoid aspect ratio approaches zero, consistent with the decay in the stress field of thin plates. Lee [44] also provided analytical solutions for elastic strain energy and interaction energy of two thin square plates with a simple shear eigenstrain. The interaction energy decreases more rapidly with increasing center distance for plates with an aspect ratio of 0.01 compared to those with an aspect ratio of 0.1. The maximum interaction energy occurs when the centers are aligned along the [001] direction and the two precipitates are nearly in contact.

The GND loop model enables us to calculate the elastic interaction energy of GP platelets, which elucidates the inclined arrangements of GP zones from a new perspective. This model can be applied to all orientational variants through coordinate transformations, allowing for the exploration of the spatial distribution of precipitates, not only for the specific orientations.

MD simulations have indicated that shearing [10], Orowan looping, and cross slip [45] occur during the interaction between dislocation and a single GP zone. PDD simulations of screw dislocation source also showed small-scale cross slip, which is not presented in this article. In addition to the lower computational cost, a key advantage of PDD simulations compared to MD is the ability to independently distinguish and study the various stress components acting on dislocation nodes. In this research, these include applied stress, stress from precipitates, and dislocation self-stress. From the perspective of elastic interactions, the strengthening effect of GP zones follows the superposition principle. Ideally, as our simulations show, non-screw dislocations uniformly shear GP zones, leading to local anisotropy but minimal global anisotropy, since the overall average internal stress remains zero in a material without residual stress. However, the cross-slip mechanism of screw dislocations and chemical hardening introduce non-uniform interactions, which in turn result in global anisotropy. While global anisotropy is indeed an interesting phenomenon, it falls outside the scope of this article, which focuses on localized edge dislocation–precipitate interactions at smaller scales.

## 5. Conclusions

In this study, we developed a line integral model of disk-shaped GP zones using concentric GND loops. The elastic fields were validated by comparing them with numerical results of oblate precipitates obtained through surface integral models based on Eshelby inclusion theory. The optimal arrangement of GP zone arrays was determined by analyzing the elastic interactions of GND loops, further supported by TEM observations. GP–dislocation interactions were explored through PDD simulations. The results revealed that 60-degree-type disks exhibit distinct strong and weak directions for dislocation approach and shearing, highlighting local anisotropy.

## Figures and Tables

**Table 1:** Nominal composition of the aluminum ingot in weight percent.

Sample	Si	Fe	Cu	Al
Al-4Cu	0.05	0.1	4.01	Balance

## Data Availability

The raw data supporting the conclusions of this article will be made available by the authors on request.
